# Automatic Grading of Stroke Symptoms for Rapid Assessment Using Optimized Machine Learning and 4-Limb Kinematics: Clinical Validation Study

**DOI:** 10.2196/20641

**Published:** 2020-09-16

**Authors:** Eunjeong Park, Kijeong Lee, Taehwa Han, Hyo Suk Nam

**Affiliations:** 1 Cerebro-Cardiovascular Disease Research Center Yonsei University College of Medicine Seoul Republic of Korea; 2 Department of Radiology Yonsei University College of Medicine Seoul Republic of Korea; 3 Health-IT Center Yonsei University College of Medicine Seoul Republic of Korea; 4 Department of Neurology Yonsei University College of Medicine Seoul Republic of Korea

**Keywords:** machine learning, artificial intelligence, sensors, kinematics, stroke, telemedicine

## Abstract

**Background:**

Subtle abnormal motor signs are indications of serious neurological diseases. Although neurological deficits require fast initiation of treatment in a restricted time, it is difficult for nonspecialists to detect and objectively assess the symptoms. In the clinical environment, diagnoses and decisions are based on clinical grading methods, including the National Institutes of Health Stroke Scale (NIHSS) score or the Medical Research Council (MRC) score, which have been used to measure motor weakness. Objective grading in various environments is necessitated for consistent agreement among patients, caregivers, paramedics, and medical staff to facilitate rapid diagnoses and dispatches to appropriate medical centers.

**Objective:**

In this study, we aimed to develop an autonomous grading system for stroke patients. We investigated the feasibility of our new system to assess motor weakness and grade NIHSS and MRC scores of 4 limbs, similar to the clinical examinations performed by medical staff.

**Methods:**

We implemented an automatic grading system composed of a measuring unit with wearable sensors and a grading unit with optimized machine learning. Inertial sensors were attached to measure subtle weaknesses caused by paralysis of upper and lower limbs. We collected 60 instances of data with kinematic features of motor disorders from neurological examination and demographic information of stroke patients with NIHSS 0 or 1 and MRC 7, 8, or 9 grades in a stroke unit. Training data with 240 instances were generated using a synthetic minority oversampling technique to complement the imbalanced number of data between classes and low number of training data. We trained 2 representative machine learning algorithms, an ensemble and a support vector machine (SVM), to implement auto-NIHSS and auto-MRC grading. The optimized algorithms performed a 5-fold cross-validation and were searched by Bayes optimization in 30 trials. The trained model was tested with the 60 original hold-out instances for performance evaluation in accuracy, sensitivity, specificity, and area under the receiver operating characteristics curve (AUC).

**Results:**

The proposed system can grade NIHSS scores with an accuracy of 83.3% and an AUC of 0.912 using an optimized ensemble algorithm, and it can grade with an accuracy of 80.0% and an AUC of 0.860 using an optimized SVM algorithm. The auto-MRC grading achieved an accuracy of 76.7% and a mean AUC of 0.870 in SVM classification and an accuracy of 78.3% and a mean AUC of 0.877 in ensemble classification.

**Conclusions:**

The automatic grading system quantifies proximal weakness in real time and assesses symptoms through automatic grading. The pilot outcomes demonstrated the feasibility of remote monitoring of motor weakness caused by stroke. The system can facilitate consistent grading with instant assessment and expedite dispatches to appropriate hospitals and treatment initiation by sharing auto-MRC and auto-NIHSS scores between prehospital and hospital responses as an objective observation.

## Introduction

Motor weakness is a typical manifestation in various neurological disorders, including stroke, spinal cord injury, and traumatic brain injury. In addition, it is a major obstacle to functional recovery after the treatment of those diseases. As an example of motor weakness, unintentional drift is an indication of arm weakness; this is mainly caused by subtle damages in the motor pathway from the brain to the spinal cord [1]. If the supinator muscles in the upper limb are weaker than the pronator muscles in the presence of upper motor neuron lesion, the arm drifts downward and the palm turns toward the floor. The pathological response is for one of the arms to drift (up, down, or out). Therefore, motor weakness is a major sign in the FAST (face drooping, arm weakness, speech slurring, and time to call) protocol for stroke patients [2].

Rapid detection of such motor weakness is critical because acute treatments, including thrombolysis or thrombectomy, are performed in a constrained time window. More importantly, diagnosis can be established through bedside examination by specialists because it is a qualitative measurement. If the symptom occurs outside a hospital, a substantial time delay can cause poor outcomes for acute stroke patients [3-5]. In addition, the objective and accurate neurological assessments are not possible by mere visual examination because the examiner cannot easily trace the movement using the conventional neurological examination when there are subtle weaknesses. Therefore, systems need to automatically detect motor deficits using sensor data in real time.

However, operating such systems in a real environment requires a significant effort in integrating new systems into an emergency protocol. This is because interruptions caused by the attachment of sensors on patients’ bodies and the initiation of the recording process can affect the streamlined structure of emergency protocols. However, evaluation methods are still required to identify stroke patients, as they can be instantly used in the communication among patients or caregivers, emergency call centers, and hospitals. In addition to a sensor-based measurement tool that was demonstrated useful in detecting subtle motor weakness in our previous study [6], the grading of stroke severity can be informed remotely and used in the emergency medical service (EMS) and hospital system.

In the field and in clinical environments, various grading methods exist for identifying ischemic stroke patients with motor weakness [7-10]. The National Institutes of Health Stroke Scale (NIHSS) score [11,12] and Medical Research Council (MRC) score [13,14] have been used as typical assessment indicators for stroke in the clinical environment. The rapid arterial occlusion evaluation scale, the Cincinnati stroke triage assessment tool, and the prehospital acute stroke severity scale are grading methods in the field environment. In this study, we implemented auto-NIHSS and auto-MRC systems to grade the NIHSS and modified MRC scores to assess patients in the clinical environment. We used subdivided MRC scores (10-grade MRC) instead of a 6-grade MRC to define subtle differences, as shown in Table 1.

**Table 1:** NIHSS and MRC grades for muscle power assessment.

Scale and grade		Description
**NIHSS^a^**		
	0	No drift; limb holds 90° (or 45°) angle for full 10 seconds
	1	Drift; limb holds 90° (or 45°) angle, but drifts down before full 10 seconds; does not hit bed or other support
	2	Some effort against gravity; limb cannot reach or maintain (if cued) 90° (or 45°) angle; drifts down to bed, but has some effort against gravity
	3	No effort against gravity; limb falls
	4	No movement
**MRC^b^**		
	0 (0)	No movement
	1 (1)	A flicker of movement is observed or felt in the muscle
	2 (1+)	Muscle moves the joint when gravity is eliminated
	3 (2)	Muscle moves the joint against gravity, but not through full mechanical range of motion
	4 (2+)	Muscle cannot hold the joint against resistance, but moves the joint fully against gravity
	5 (3)	Muscle moves the joint fully against gravity and is capable of transient resistance, but collapses abruptly
	6 (3+)	Same as grade 4 (on 6-point scale) but muscle holds the joint only against minimal resistance
	7 (4)	Muscle holds the joint against a combination of gravity and moderate resistance
	8 (4+)	Same as grade 4 (on 6-point scale) but muscle holds the joint against moderate to maximal resistance
	9 (5)	Normal strength

^a^NIHSS: National Institutes of Health Stroke Scale.

^b^MRC: Medical Research Council.

## Methods

### Participants and Data

A total of 17 participants were recruited; 15 participants (10 male and 5 female participants) were finally enrolled and completed 4-limb drift test trials. To estimate the scores of patients with severity, we performed the assessment shortly after admission to a stroke unit. The ages of the participants ranged from 44 to 92 years, with a mean of 68.6 years (SD 16.11). Exclusion criteria were patients (1) who had a substantial weakness that prevented arm or leg raising against gravity, (2) who were not able to sit and who had bilateral arm weakness or preexisting chronic arm weakness, and (3) who had aphasia, neglect, peripheral neuropathy, myopathy, or joint deformity. This study was approved by the Severance Hospital Institutional Review Board, and informed consent was obtained from all participants.

**Figure 1:**
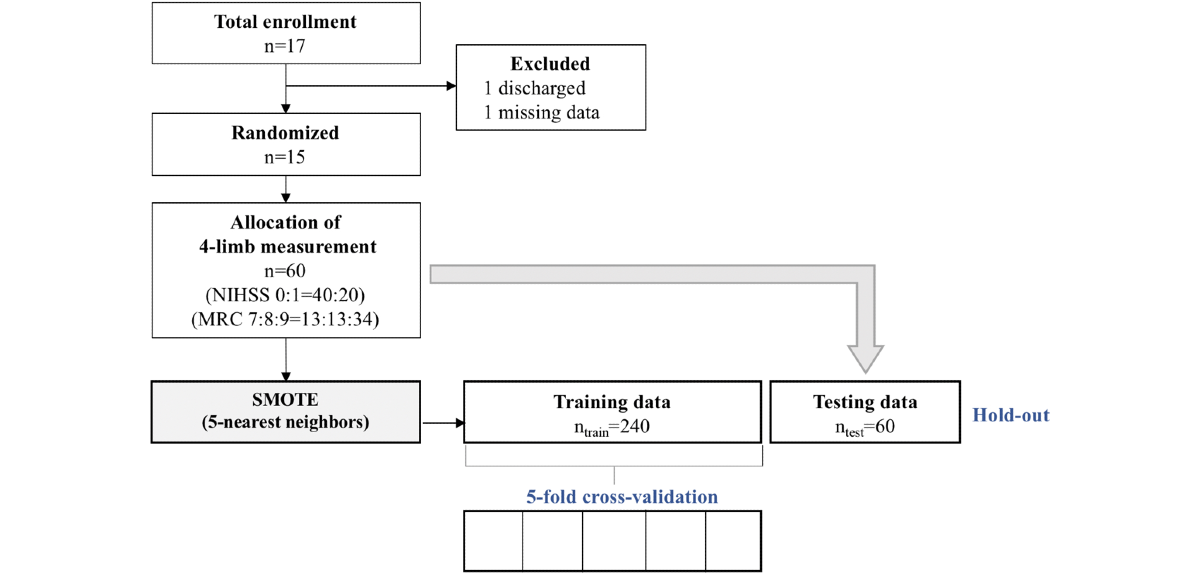
Patient enrollment and data set for automatic grading system. MRC: Medical Research Council; NIHSS: National Institutes of Health Stroke Scale; SMOTE: synthetic minority oversampling technique.

Figure 1 shows patient enrollment and data preparation for auto-NIHSS and auto-MRC grading. Description of data composition for training, validation and testing is detailed in the section on system design.

### System Design

**Figure 2:**
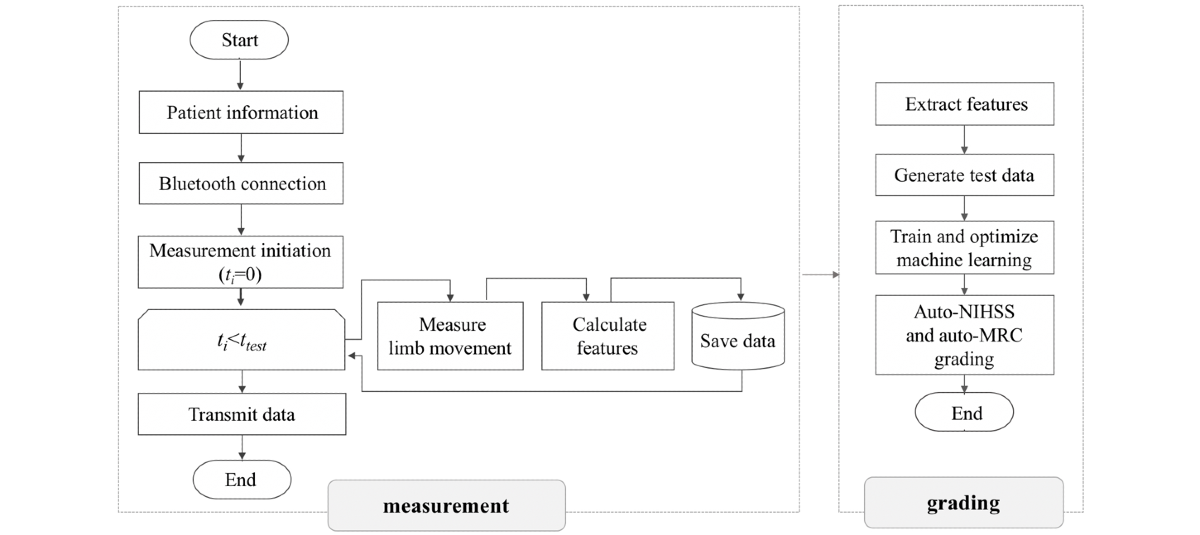
Automatic grading process. MRC: Medical Research Council; NIHSS: National Institutes of Health Stroke Scale.

The entire process of the system is shown in Figure 2. The system is composed of 2 parts, the measurement and the grading units. The measurement unit sets up sensors and Bluetooth connection with the primary information of patients.

**Figure 3:**
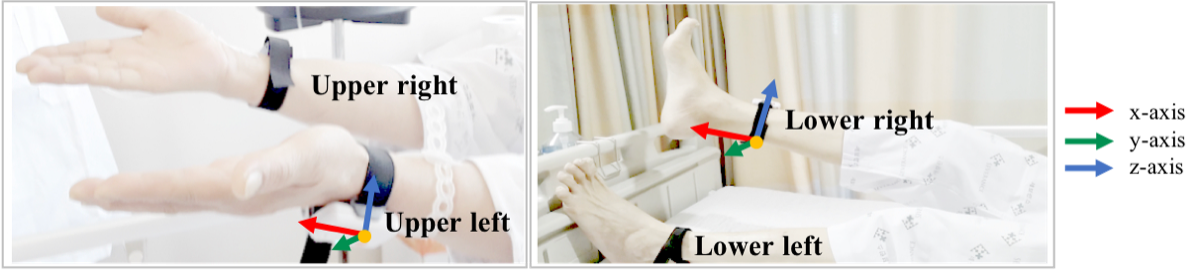
Schematic of upper and lower limb sensors and corresponding segment axes.

We measured the upper left and upper right limb movements using sensors on both wrists of patients, who were asked to stretch and hold their arms for 20 seconds, as shown in Figure 3. For the lower left and lower right limb drift tests, patients were asked to lift and stretch their left or right leg for 20 seconds.

The pseudo-code of the measurement unit is shown in Multimedia Appendix 1. For each time frame *i*, the rotational transformation from the limb into the reference frame *xyz* is denoted as 
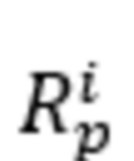
. The corresponding rotation matrices *R* for each angle are defined using the 
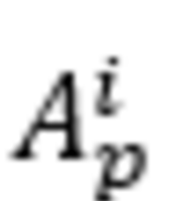
 of the accelerometer signals for the *i*th frame. Subsequently, the degree of drift, θ*_drift_*, is calculated and used in key features of machine learning classification.

After collecting the series of 4-limb movements during the test time, the grading unit analyzes the kinematic features. Subsequently, the machine learning algorithm is trained to estimate the NIHSS and MRC scores of each limb. Algorithm 2 (in Multimedia Appendix 2) shows the process of feature extraction, data generation, and model training for the optimized classification of auto-NIHSS and auto-MRC.

In the feature extraction process, features as predictors of limb paralysis were extracted using a series of measured data. In this study, the duration of the drift test (*t_test_*) was set to 20 seconds; however, analysis started 10 seconds after the examination started (*t_start_*) to exclude the initial dip. The average, maximum, and oscillation of drift caused by paralysis for each limb and demographic features were fed to train the machine learning algorithms.

In the data generation process, we adopted the synthetic minority oversampling technique (SMOTE) [15], leveraging the K-nearest neighbor (K-NN), to solve the imbalanced problem that is typical in machine learning studies in medicine [16-18]. The SMOTE with K-NN generated *n_g_* samples for each grade. Therefore, *n_g_c* records were used to construct a grading model with *c* classes. In this study, *n_g_* was set to 120 for auto-NIHSS (*c*=2) and 80 for auto-MRC (*c*=3) to compose the training data with 240 (*t_train_*) instances. Apart from the training data, the original data set with 60 records remained for the test data, as shown in Figure 1.

In the training process, 5-fold cross-validation was applied to reduce overfitting and generalize the model [19]. In the optimization process, the fitted support vector machine (SVM), as well as ensemble models among various SVM kernels and boosting algorithms with tuned hyperparameters, were searched via Bayes optimization in 30 trials for each model [20]. The grading models were implemented and evaluated in MATLAB R2020a (MathWorks Inc) [21].

## Results

### Sensor Data Characteristics

**Figure 4:**
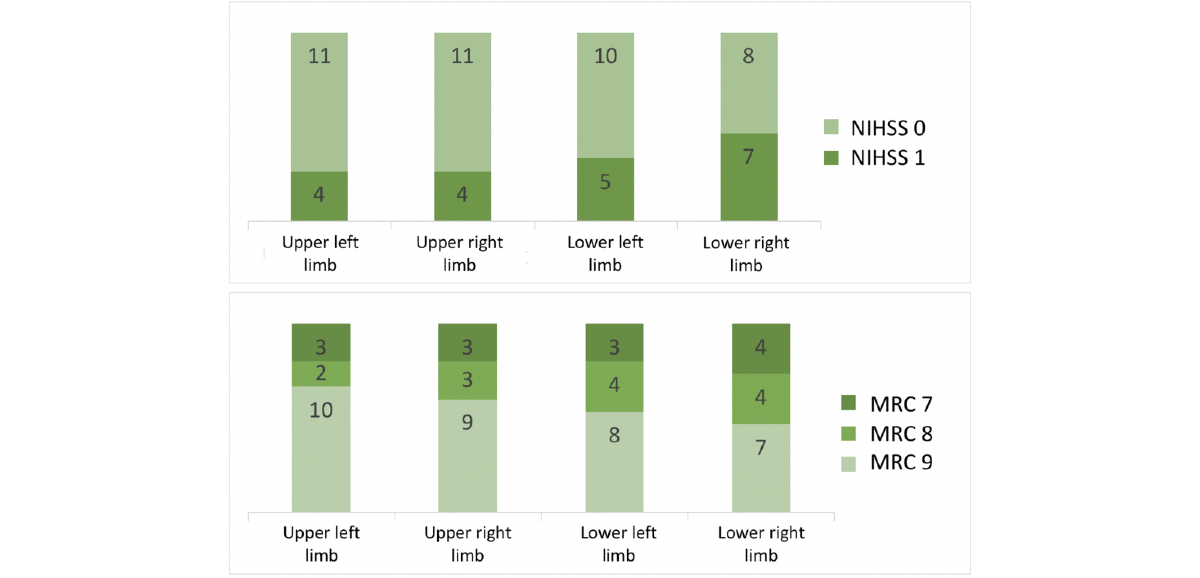
Grade distribution of NIHSS and MRC. MRC: Medical Research Council; NIHSS: National Institutes of Health Stroke Scale.

The system measured the drift of 4 limbs and extracted the kinematic features, as shown in Multimedia Appendix 3. The characteristics of the patients and test data are summarized in Table 2. The grade distribution of clinical scores was not regularized between limbs, as shown in Figure 4. For example, the upper left MRC group had 10 patients graded as MRC 9, 2 patients graded as MRC 8, and 3 patients graded as MRC 7. Among 13 MRC 8 instances, 7 were evaluated as NIHSS 1, whereas 6 were evaluated NIHSS 0. We constructed auto-MRC, which discriminated instances of grades with a data ratio of 13:13:34, whereas auto-NIHSS performed binary classification with a data ratio of 40:20.

**Table 2:** Summary of patients and test data.

Diagnosis	Measurement																NIHSS^a^ grade (MRC^b^ grade)						
	ULL^c^				URL^d^				LLL^e^				LRL^f^				ULL		URL		LLL		LRL
	Mean	Max^g^	Osc^h^	Mean		Max	Osc	Mean		Max	Osc	Mean		Max	Osc								
Lt^i^ internal capsule infarction	0.82	2.7	14.4	–3		–1.9	15.3	–1.19		2.1	25.1	11.81		17.4	30.8	0 (9)		0 (8)		0 (9)		1 (8)	
Lt MCA^j^ infarction	–9.33	–12	15.9	–6.47		–9.2	11.7	18.7		10.7	43.5	30.26		27.7	13.6	0 (8)		1 (7)		0 (8)		1 (7)	
Lt MCA infarction	0.86	0	7	4.06		1.4	13.8	2.96		0	24.9	9.91		–1.6	61.5	0 (9)		0 (9)		0 (9)		1 (7)	
Lt MCA infarction	3.16	4.2	14.5	2.3		3.2	19.1	0.26		1.6	12.9	4.26		8.4	21.7	0 (9)		0 (9)		0 (9)		0 (8)	
Lt MCA infarction	1.92	3.6	14.6	3.14		4.2	14.5	1.84		5.7	39.5	0.75		2.9	19.6	0 (9)		0 (9)		0 (9)		0 (9)	
Lt pontine infarction	–0.67	0.6	19.5	–1.37		1.3	12.9	–11.93		–10.3	16.7	–4.93		–2.1	17.3	1 (7)		0 (8)		1 (7)		1 (8)	
Lt thalamic infarction	2.05	3.5	22.8	8.91		11.4	12.5	4.77		8.8	31.3	1.98		6.8	37.7	0 (9)		1 (7)		0 (9)		1 (8)	
Pontine ICH^k^	–1.57	1.5	39.1	0.81		2	18.5	–3		1.2	40	3.18		5.3	16.5	1 (7)		0 (9)		1 (8)		0 (9)	
Rt^l^ MCA infarction	–9.96	–7.5	17.9	–1.93		–0.6	19	–2.71		0.4	18.5	–1.99		–0.3	17.2	1 (7)		0 (9)		1 (7)		0 (9)	
Lt internal capsule infarction	–6	–7.9	14	–0.8		–2	11.6	1.8		0.8	18.6	11		6.5	38.5	0 (9)		0 (9)		0 (8)		0 (9)	
Myelitis (no weakness)	1.3	2.9	18.6	–0.56		0.1	11.7	–1.23		1.2	24.1	–1.14		0.7	24	0 (9)		0 (9)		0 (9)		0 (9)	
Rt MCA infarction	–4.97	–6.4	19.2	0.7		0	13.1	13.9		7	49.3	6.31		2.3	34.3	0 (9)		0 (9)		1 (7)		0 (9)	
Myasthenia gravis	–0.64	1.3	19.2	1.1		2.7	14.4	–1.97		0	18.5	–0.64		2.7	22.6	0 (9)		0 (9)		0 (9)		0 (9)	
Lt pontine infarction	15.5	5.4	41.1	23.5		12	54	6.3		2.2	26.1	5.3		0.6	46.2	0 (9)		1 (7)		0 (9)		1 (7)	
Pontine hemorrhage	–0.83	1.1	19	–2.72		1.3	26.6	1.69		3.3	13.6	–7.52		–0.8	54.5	1 (8)		1 (8)		1 (8)		1 (7)	

^a^NIHSS: National Institutes of Health Stroke Scale.

^b^MRC: Medical Research Council.

^c^ULL: upper left limb.

^d^URL: upper right limb.

^e^LLL: lower left limb.

^f^LRL: lower right limb.

^g^Max: maximum.

^h^Osc: oscillation.

^i^Lt: left.

^j^MCA: middle cerebral artery.

^k^ICH: intracerebral hemorrhage.

^l^Rt: right.

### Evaluation Outcomes

We evaluated the performance of the system in terms of the accuracy, sensitivity, specificity, precision, F1 score, and area under the receiver operating characteristics curve (AUC) with a confusion matrix.

**Figure 5:**
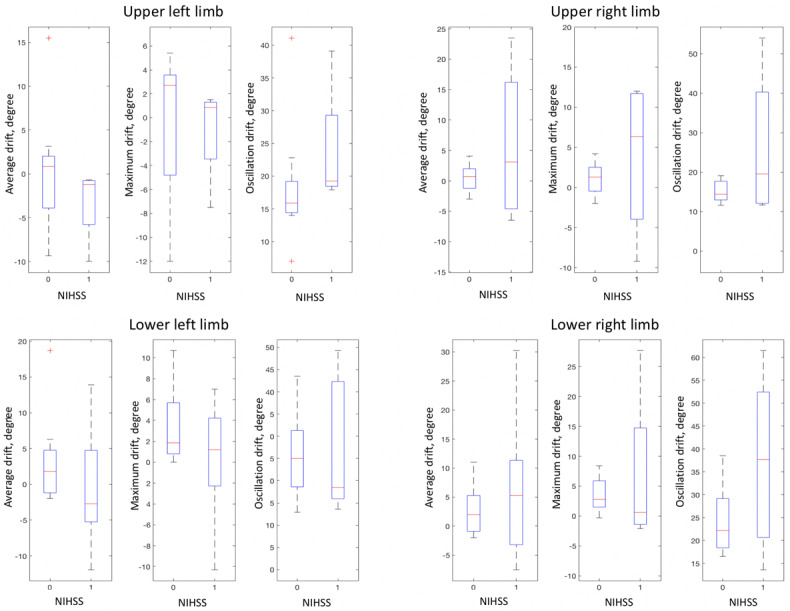
Statistical plots of 4-limb features of NIHSS grades. NIHSS: National Institutes of Health Stroke Scale.

**Figure 6:**
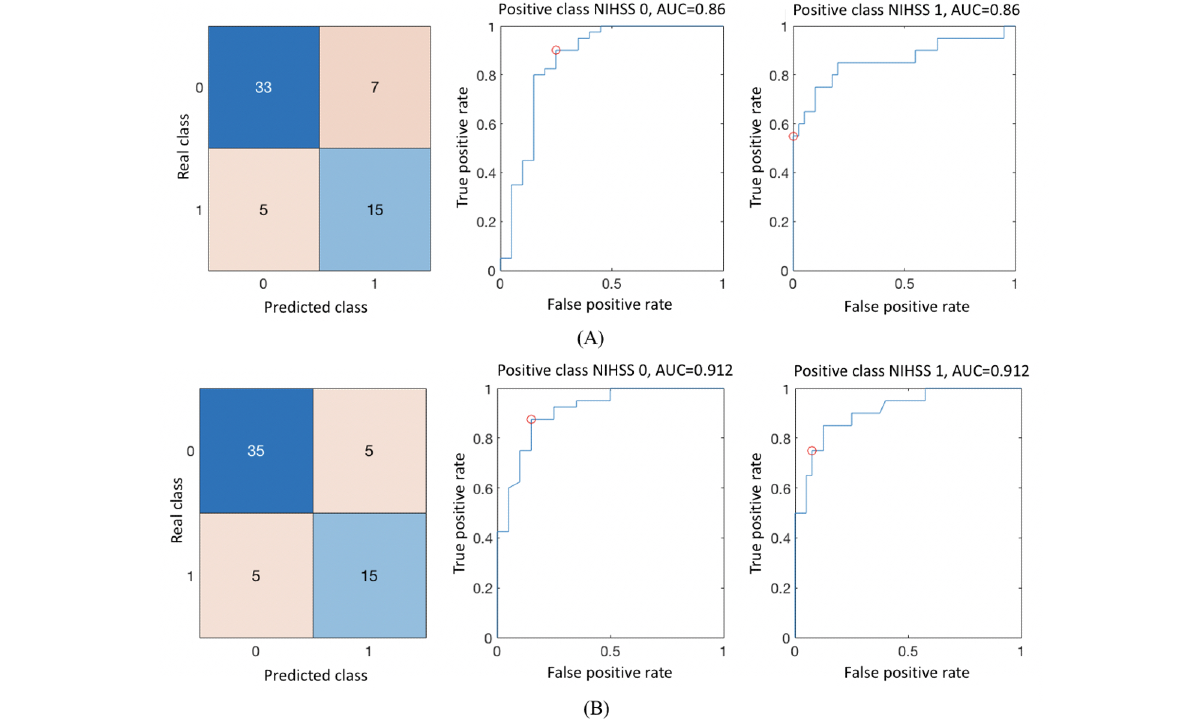
Confusion matrix and receiver operating characteristic of auto-NIHSS grading using (A) support vector machine and (B) ensemble learning. AUC: area under the receiver operating characteristics curve; NIHSS: National Institutes of Health Stroke Scale.

The statistical plots in Figure 5 show the patterns of the average, maximum, and oscillation of the 4-limb features of each NIHSS grade. Auto-NIHSS discriminated those features, as shown in the confusion matrices in Figure 6. The result shows that the proposed autonomous grading achieved an accuracy of at least 80% and that the overall accuracy was 81.7%, as shown in the summary of performance in Table 3. The AUC of auto-NIHSS reached 0.912, as depicted in the receiver operating characteristics curves in Figure 6. The sensitivity of the NIHSS grading reached 0.825 with the SVM and 0.875 with the ensemble. The specificity was 0.750 for both models.

**Figure 7:**
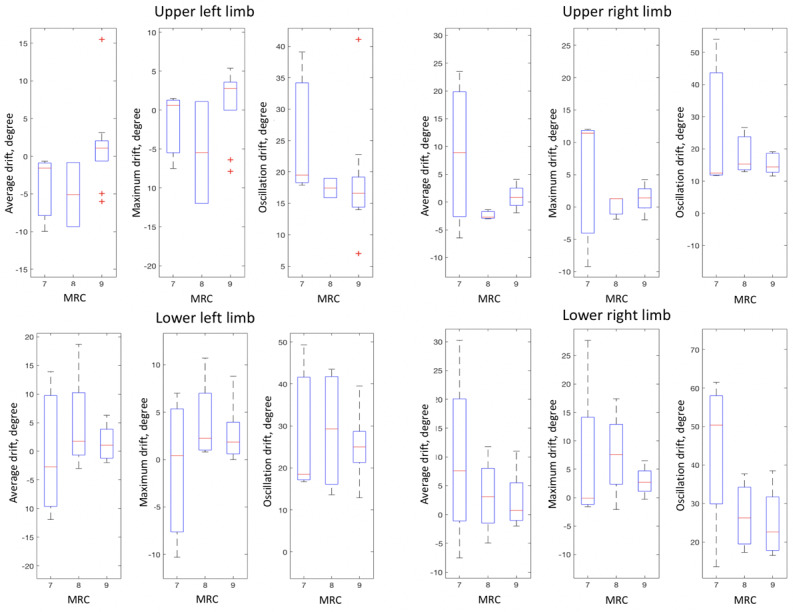
Statistical plots of 4-limb features of MRC grades. MRC: Medical Research Council.

**Figure 8:**
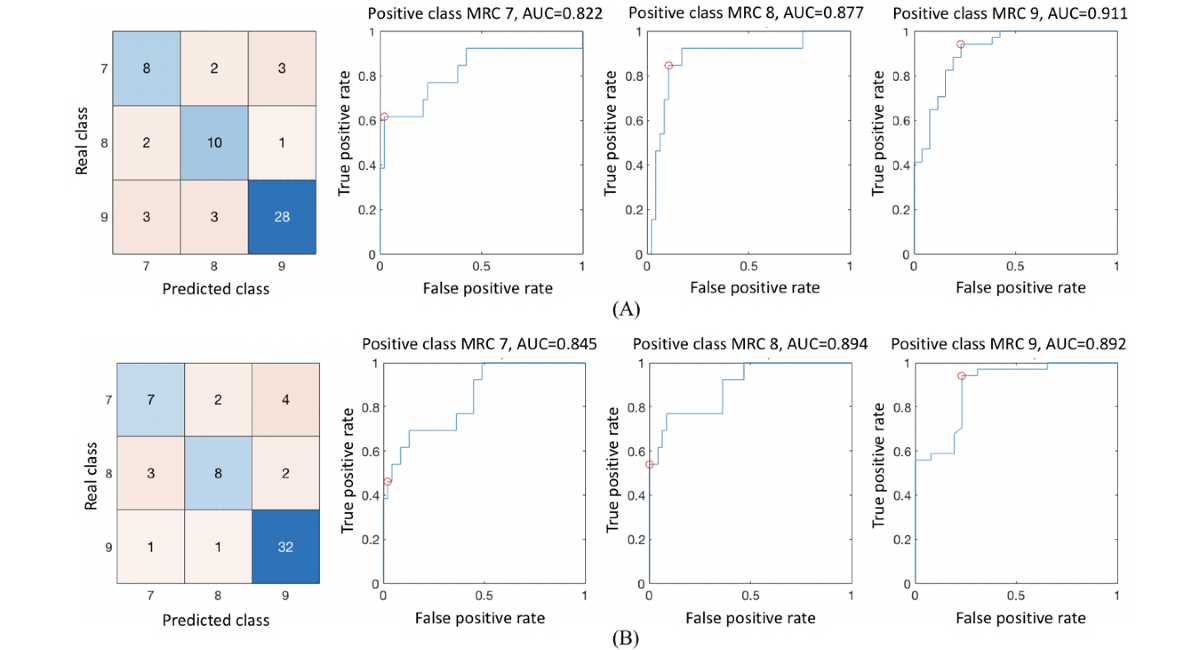
Confusion matrix and receiver operating characteristic of auto-MRC grading using (A) support vector machine and (B) ensemble learning. AUC: area under the receiver operating characteristics curve; MRC: Medical Research Council.

Auto-MRC discriminates instances into 3 MRC grades, and the statistical plots of movement features are depicted in Figure 7. The mean AUC was 0.870 for the SVM and 0.877 for the ensemble, as shown in Figure 8. Table 4 shows the summarized performance of auto-MRC; the average accuracy, sensitivity, and specificity for the MRC grading were 0.775, 0.717, and 0.876, respectively.

**Table 3:** Performance of auto-NIHSS grading.

Auto-NIHSS^a^ grading	Accuracy	Sensitivity	Specificity	Precision	F1 score
SVM^b^	0.800	0.825	0.750	0.868	0.846
Ensemble	0.833	0.875	0.750	0.875	0.875

^a^NIHSS: National Institutes of Health Stroke Scale.

^b^SVM: support vector machine.

**Table 4:** Performance of auto-MRC grading.

Auto-MRC^a^ grading	Accuracy	Sensitivity	Specificity	Precision	F1 score
SVM^b^	0.767	0.736	0.878	0.719	0.726
Ensemble	0.783	0.698	0.873	0.735	0.713

^a^MRC: Medical Research Council.

^b^SVM: support vector machine.

## Discussion

### Importance of Objective and Fast Assessment of Stroke Severity

The notion “time is brain” is valid in treating stroke patients. Intravenous tissue plasminogen activator (IV tPA) within 4.5 hours of stroke onset is the only therapy for acute ischemic stroke [22]. Subsequently, endovascular thrombectomy (EVT) has been a standard of care for patients with acute ischemic stroke caused by large artery occlusion within 6 to 24 hours of onset, based on successful large randomized clinical trials [23]. Reperfusion therapy, including IV tPA and EVT, for acute ischemic stroke is time sensitive (ie, an earlier treatment yields a better outcome). As the onset-to-intervention time is composed of prehospital and in-hospital phases, patients who arrive early have more chances of appropriate treatment [24-27]. Delays in hospital admission and the preparation before treatment affect the prognosis of patients [28]. In Goyal et al [24], the authors reported that the most significant issue was getting the correct patient to the correct hospital quickly. In Sukumaran et al [27], strategies for stroke patient workflow optimization were suggested by analyzing and solving prehospital and preprocedural bottlenecks. The interhospital transfer is directly associated with delays in onset to reperfusion time, which results in the poor outcome of stroke patients; therefore, the timely triage of patients is a significant bottleneck [27].

The importance of accurate and objective assessments of stroke severity in telemedicine and telestroke strategies has been discussed in numerous studies [29]. In particular, the timing constraint in performing reperfusion therapy, which has been shown to significantly reduce mortality, invokes the development of efficient systems and protocols in prehospital care or emergency medical systems. Researchers have addressed the fact that the rapid and accurate evaluation of stroke severity can aid in identifying patients for treatments and accelerate an urgent streamlined process. In the study by Andsberg et al [30], a prehospital ambulance stroke test was performed to score the severity of stroke through commands, answers, and observations. The remote assessment of stroke using smartphones was proposed and compared with bedside examination in calculating the NIHSS score [31]. However, most assessments in those systems used conservative observation or campaigns that were subjective and unreliable between testers. Modern communication, sensor technology, and machine learning can solve this problem through accurate measurements and the fast determination of assessment in a prehospital or remote environment [29,32,33]. A previous study evaluated arm function in activities using kinematic exposure variation analysis and inertial sensors [34]. A mobile-based walk test was developed to report patients’ walking ability [35], and upper limb impairments in stroke patients were measured using inertial sensors in the home environment [33]. Such sensor-based testing enables objective evaluation regardless of the testers or place.

### Utility of Consistent Grading Method as an Agreement Between Prehospital and Hospital Environment

The necessity of a controlled test is revealed in the results of previous studies for monitoring daily living. Motor recovery was monitored using accelerometers, and the NIHSS motor index was estimated in the study by Gubbi et al [36]. However, the movement in daily living limited the accuracy of estimation to 56% for the low index. Activity monitoring in most sensor-based studies involved trials that were not approved by clinical protocols. Those systems limited extensibility as a standard of remote monitoring systems, although they were efficient in tracking the progress or the treatment outcome.

In addition to rapid and accurate measurements, we aimed to increase the utility of the assessment system in the prehospital and hospital environments. At every phase of the prehospital process, consistent methods to conduct assessments can reduce errors and delays in communication among the participants of a community’s emergency group. Therefore, automatic scoring can facilitate agreement in assessments among patients, caregivers, paramedics, and medical staff. With regard to bottleneck analysis in acute stroke treatment, the rapid identification of neurological deficits and assessment of motor grading will aid EMS personnel in transporting patients to a comprehensive stroke center because hospitals may be limited in terms of stroke unit availability and resources. In Berglund et al [26], the importance of stroke identification without meeting the patient or without neurological examination was asserted; the time to treatment can be decreased with the high-priority dispatch of ambulances through early identification of stroke from emergency calls. In the hyper acute stroke alarm study [25], researchers observed that higher prehospital priority levels of stroke improved thrombolysis frequency and time to stroke unit. The stroke identification by EMS dispatchers during emergency calls varied between 31% and 57%, as identifying stroke can be a challenge without examination [26].

Therefore, we developed an automatic grading system, leveraging multiclassification of machine learning using typically performed tests and grading in clinics. Our proposed solution uses controlled observations of drift tests in clinics and can estimate the assessment by neurologists. Therefore, the scores by the automatic grading system can be instantly used for communication in an objective manner.

### Data and Techniques for Clinical Scoring by Machine Learning

A considerable number of studies have used artificial intelligence, including machine learning, to estimate clinical scores and assess patients or provide warnings regarding adverse events [37-40]. In those studies, a series of various techniques were used according to the scale of scores, the capacity of collected data, and the skewness of data. Following the significant development of enhanced algorithms, data with significant meaning have gained importance. However, as addressed in Li et al [41], real-world data have a long-tail pattern with a significant imbalance in quality and quantity. Many algorithms have used public big data to develop new algorithms and build models; however, real-world applications have completely different data quality and quantity and cannot directly apply those models. This situation is particularly severe in medicine, as discussed in Hulsen et al [42]. The availability of qualified data differs by disease, severity of disease in patients, and difficulty of collection [43]. Big data from electronic medical records that are already facilitated in hospital information systems can be used in comparatively easy tasks for medical artificial intelligence. The recent success of medical artificial intelligence requires significant effort and cost in collecting and labeling data [44,45]. In addition, machine learning for sporadic events in emergencies or patients with rare diseases is affected by data deficiency. This is because interventions for collecting data can affect the prognosis of treatment due to the possible delay in the rapid streamlining of treatment processes. Previous feasibility studies have stated that the difficulty in real-time capturing of acute neurological disorders was the main limitation in the research [33,46].

The learning models with imbalanced data were affected by low precision or recall in the validation and test phases, although they achieved high accuracy for a large number of data in the majority groups [47]. Recently, techniques to solve this data skewness, including data augmentation, transfer learning, and deep imbalanced learning, were emphasized [48-51]. Studies on deep learning that extract filtered features derived from raw data have attempted to solve the problem by knowledge transfer from pretrained models [52,53] or with data augmentation [54,55]. Machine learning with records can cope with the imbalance problem through sampling, cost-sensitive learning, boosting algorithms, and skew-related performance metrics [47,56]. We used the SMOTE to balance between classes in the training phase and applied techniques, including RUSBoost, in optimized ensemble machine learning. To compare different models according to their precision on each class, the F measure is typically used as a performance metric [57]; additionally, we validated the performance of the proposed solution using the AUC and F1 scores. Consequently, the performances of auto-NIHSS and auto-MRC indicated the acceptable AUC, sensitivity, specificity, and F1 score as real-world applications with data skewness.

### Conclusion

Accurate monitoring and grading of motor weakness are critical for the appropriate assessment of stroke severity, particularly for reliable and consistent evaluations. We developed an automatic grading system to assess proximal motor weakness using the kinematic features of unintended drift of 4 limbs. We trained optimized machine learning models and obtained promising results in scoring NIHSS and MRC. The objective scoring of neurological deficits can be used to identify stroke patients, dispatch patients to the appropriate medical center, and expedite treatment preparation.

## Appendix

Multimedia Appendix 1 Algorithm of measurement unit for automatic grading.

Multimedia Appendix 2 Algorithm of the grading unit for extracting features and training machine learning algorithms. MRC: Medical Research Council; NIHSS: National Institutes of Health Stroke Scale; SMOTE: synthetic minority oversampling technique.

Multimedia Appendix 3 Sample measurement of unintended drift of limbs.

## Abbreviations

AUC: area under the receiver operating characteristic curve
EMS: emergency medical service
EVT: endovascular thrombectomy
IV tPA: intravenous tissue plasminogen activator
K-NN: K-nearest neighbor
MRC: Medical Research Council
NIHSS: National Institutes of Health Stroke Scale
SMOTE: synthetic minority oversampling technique
SVM: support vector machine
