# Supplementary figures and images for: Automatic Grading of Stroke Symptoms for Rapid Assessment Using Optimized Machine Learning and 4-Limb Kinematics: Clinical Validation Study
Source: J Med Internet Res. 2020 Sep 16;22(9):e20641. doi: 10.2196/20641 (PMC7527905; doi:10.2196/20641)

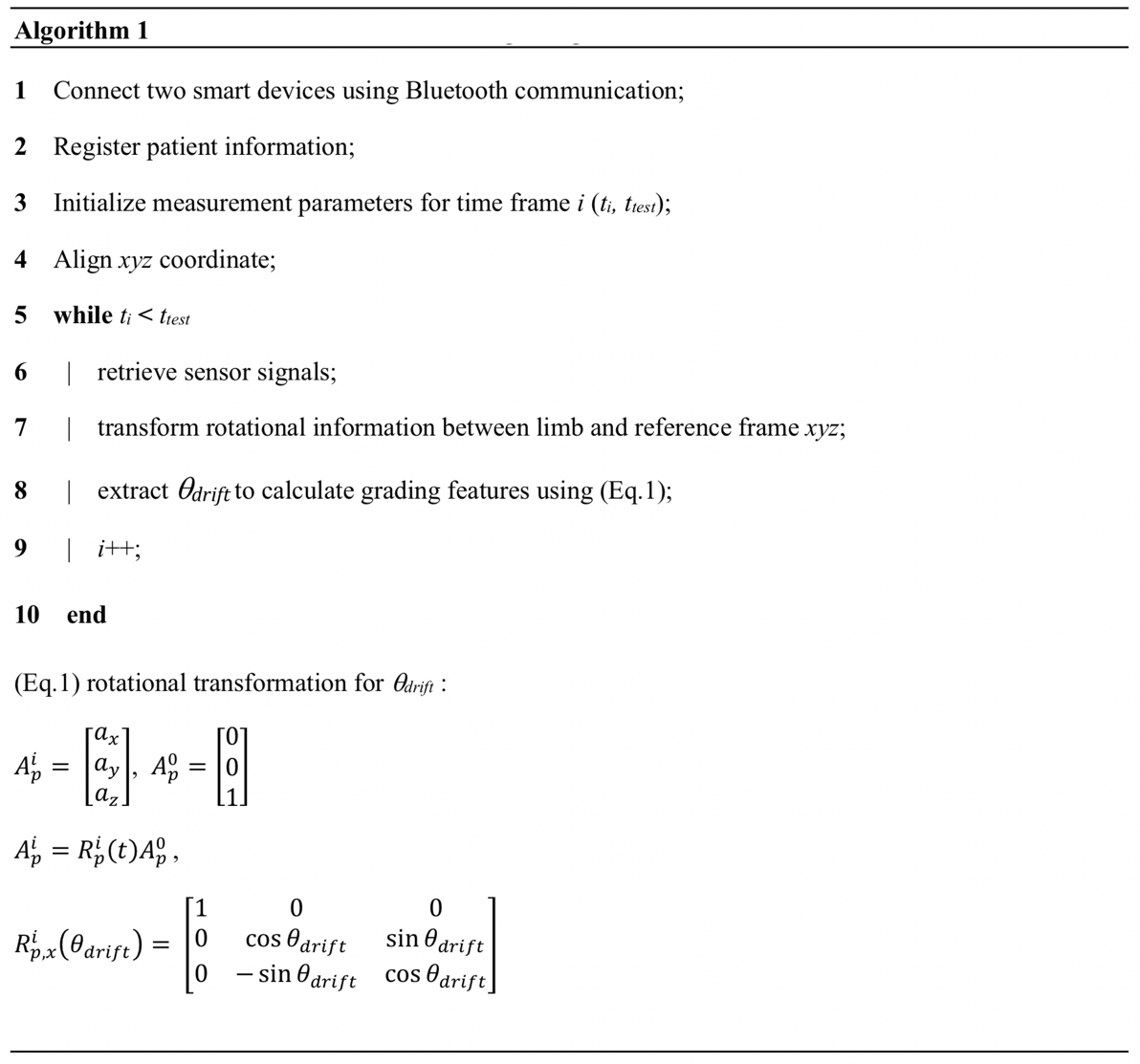

Supplement: Multimedia Appendix 1 [file jmir_v22i9e20641_app1.png]

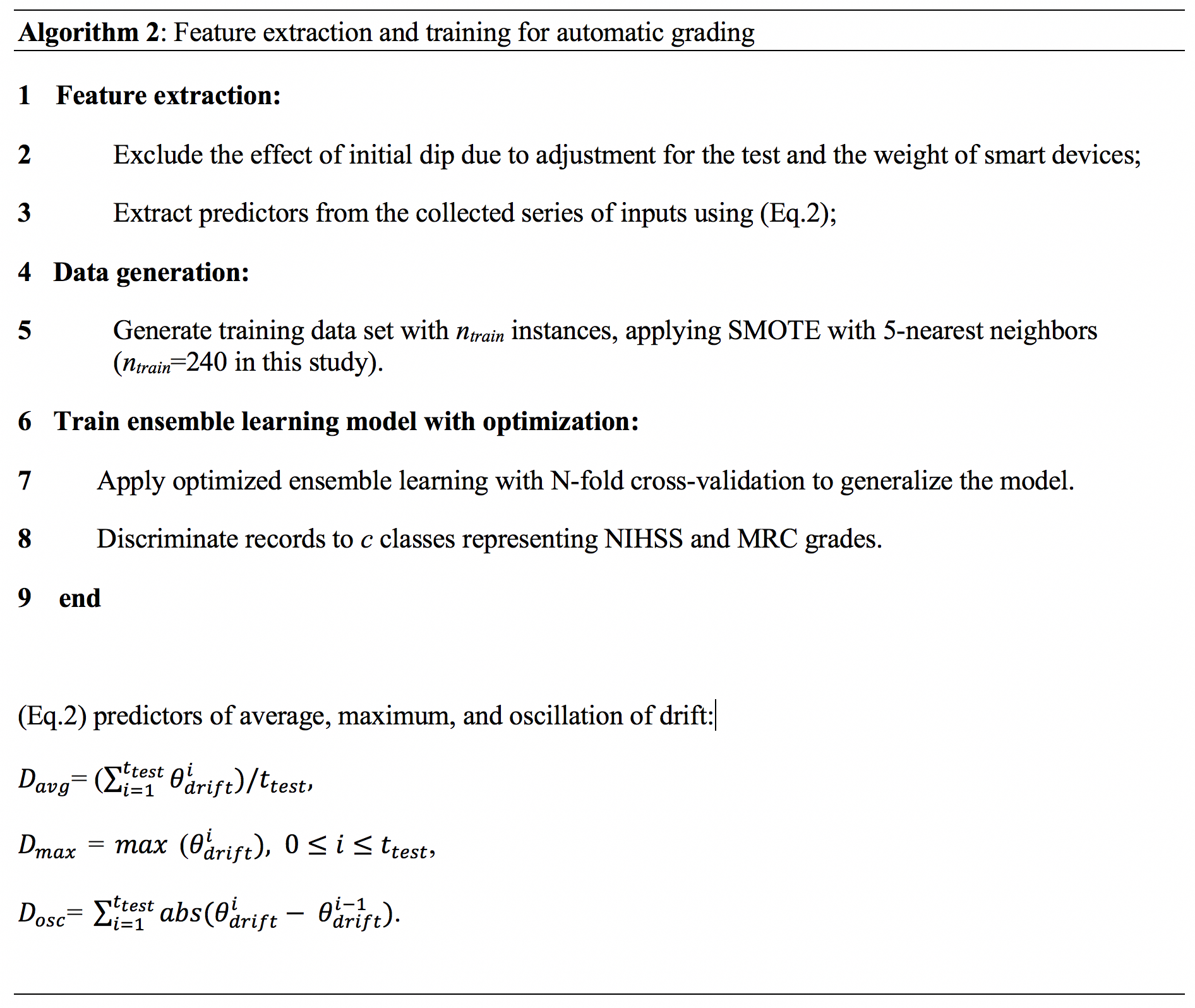

Supplement: Multimedia Appendix 2 [file jmir_v22i9e20641_app2.png]

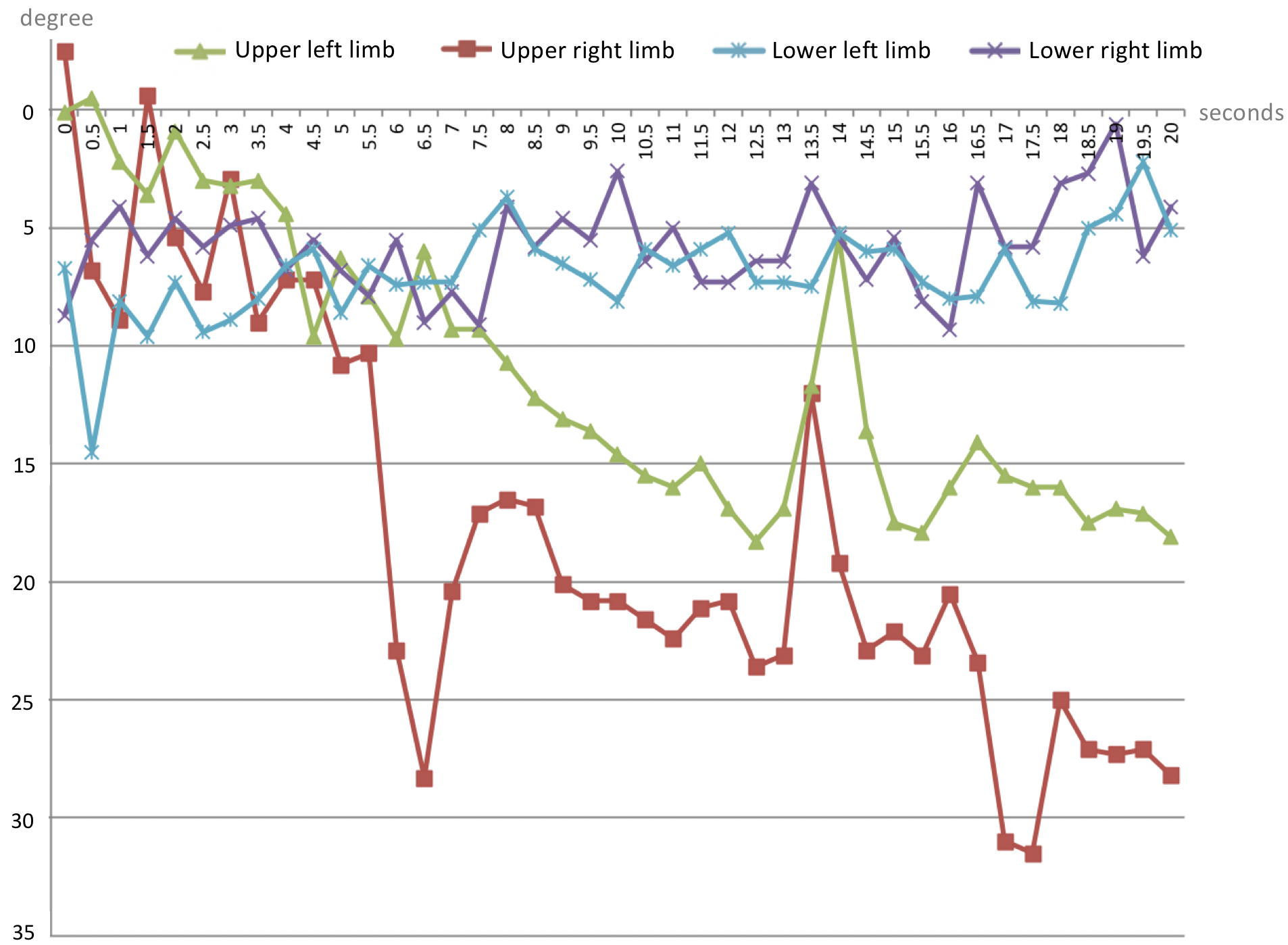

Supplement: Multimedia Appendix 3 [file jmir_v22i9e20641_app3.png]
